# A Survey of Current Resources to Study lncRNA-Protein Interactions

**DOI:** 10.3390/ncrna7020033

**Published:** 2021-06-08

**Authors:** Melcy Philip, Tyrone Chen, Sonika Tyagi

**Affiliations:** 1 School of Biological Sciences, Monash University, 25 Rainforest Walk, Clayton, VIC 3800, Australia; melcy.p.j@gmail.com (M.P.); Tyrone.Chen@monash.edu (T.C.); 2 Monash eResearch Centre, Monash University, Clayton, VIC 3800, Australia; 3 Department of Infectious Disease, Monash University (Alfred Campus), 85 Commercial Road, Melbourne, VIC 3004, Australia

**Keywords:** LPI, lncRNA, ncRNA, protein, transcriptomics, molecular docking, machine learning, deep learning, databases

## Abstract

Phenotypes are driven by regulated gene expression, which in turn are mediated by complex interactions between diverse biological molecules. Protein–DNA interactions such as histone and transcription factor binding are well studied, along with RNA–RNA interactions in short RNA silencing of genes. In contrast, lncRNA-protein interaction (LPI) mechanisms are comparatively unknown, likely directed by the difficulties in studying LPI. However, LPI are emerging as key interactions in epigenetic mechanisms, playing a role in development and disease. Their importance is further highlighted by their conservation across kingdoms. Hence, interest in LPI research is increasing. We therefore review the current state of the art in lncRNA-protein interactions. We specifically surveyed recent computational methods and databases which researchers can exploit for LPI investigation. We discovered that algorithm development is heavily reliant on a few generic databases containing curated LPI information. Additionally, these databases house information at gene-level as opposed to transcript-level annotations. We show that early methods predict LPI using molecular docking, have limited scope and are slow, creating a data processing bottleneck. Recently, machine learning has become the strategy of choice in LPI prediction, likely due to the rapid growth in machine learning infrastructure and expertise. While many of these methods have notable limitations, machine learning is expected to be the basis of modern LPI prediction algorithms.

## 1. Introduction

Transcriptomics is the study of a complete set of RNA transcripts in a cell, measuring variable expression levels of the genome under different conditions. Modern transcriptomics is performed with high-throughput sequencing to investigate the function of genes and biological pathways, commonly with bioinformatics methods applying differential gene expression analyses, splice site identification, transcript variant identification or determining alternative promoter usage for protein-coding transcripts [1]. However, these protein-coding transcripts only represent a small proportion of the transcriptome. A large proportion of the genome generates RNA transcripts which do not directly code for protein products [2]. These non-coding RNA (ncRNA) transcripts have been known to exist, but their properties make them difficult to characterise as compared to the coding transcripts. ncRNA can be divided into multiple categories based on function and length [3]. In this review, we specifically consider the long non-coding RNA (lncRNA) category of ncRNA and their interaction with proteins, an important functional mechanism of lncRNA.

LncRNA are very broadly defined as RNA transcripts exceeding 200 nucleotides (nt) in length without coding potential. Their length varies widely, ranging from hundreds to thousands of nucleotides [4]. LncRNA can act as gene regulators, and like other epigenetic mechanisms are involved in numerous biological processes. They achieve their regulatory function with their ability to interact with a wide range of biological molecules, such as other nucleic acids and proteins [5], as well as with small molecules [4]. Among their more direct modes of action are sequestering and releasing transcripts to modulate gene expression, stabilising transcripts and binding to DNA to sterically hinder transcription initiation [6]. More indirectly, they can recruit proteins and other molecules to form a functional complex, or act as a scaffold for targeted chromatin formation [7].

An important layer of lncRNA-mediated gene regulation is LPI (lncRNA-protein interactions). We illustrate the importance of LPI in developmental and abiotic stress pathways with several examples encompassing multiple distinct species. In *Drosophila melanogaster*, regulatory networks mediated by LPI regulate key eye development [8] and dosage compensation pathways [9] mediated by RNA-binding proteins. In the plant *Arabidopsis thaliana*, LPI control alternative splicing within the nucleus by selectively displacing existing transcripts and subsequently altering root development [10,11]. Response to abiotic stress is also governed by LPI, as shown by an lncRNA recruiting histone methylases to suppress *Arabidopsis thaliana* flowering during cold conditions [12]. *Dario renio* LPI are also observed to interface with transcription factors and other RNA-binding proteins during embryonic development, although their exact mechanism of action is not well known [13]. LPI also act as mediators of other epigenetic mechanisms, for instance as chromatin scaffolds to organise the three-dimensional structure of the genome in *Mus musculus* [14].

Due to the widespread involvement of LPI in epigenetics, dysregulation of certain LPI contributes to disease states, particularly cancers. Severity of a human pancreatic cancer phenotype is driven by an lncRNA-protein complex, which triggers a positive feedback loop of protein overexpression leading to poor patient outcomes [15]. Similarly, formation of an lncRNA-protein complex is associated with poorer prognosis in breast cancer [16], colon cancer [16] and lymphoma [17] by blocking phosphorylation sites, stabilising other epigenetic factors and through an unknown mechanism, respectively. Infectious diseases are also associated with LPI dysregulation, including COVID-19 [18,19]. A more exhaustive list of known LPI–disease associations is available at the LncTarD database [20]. Despite the wealth of information on LPI–disease associations, their precise mechanism of action remains unknown. Therefore, insight into LPI will be valuable in complex disease research, potentially resulting in improved diagnosis and treatment procedures.

Multiple high-throughput laboratory assays were developed to investigate LPI, some of which will be briefly discussed in this review article. However, exhaustively performing an experimental validation for each individual LPI is not practical given their volume and variety. Hence, computational methods are necessary to screen these high-throughput assays for potential LPI which can then be subsequently experimentally validated, similar to transcriptomics workflows for conventional protein-coding RNA [21]. A variety of these computational LPI predictors exist, each applying different strategies to achieve their goals, and are dependent on a few biological databases containing subsets of experimentally validated LPI. In this review, we will discuss recent bioinformatics resources for studying LPI, with an emphasis on software and databases, together with their advantages as well as limitations.

## 2. LPI Laboratory Assays

Because of the biological importance of LPI, many laboratory assays were developed to identify these interactions. Two general categories of such assays exist, protein-centric assays and RNA-centric assays, which can capture either the cellular environment of a living cell or extracted biological material [22]. Protein-centric assays target the protein component of a LPI, while RNA-centric assays target the lncRNA component. Each method varies in sensitivity and specificity, has different prerequisites and has unique advantages as well as disadvantages. Comprehensively comparing and contrasting these laboratory assays is out of the scope of this review, but we provide a high-level overview only to give the computational methods discussed in this article some biological context. A more detailed overview of these assays can be found in a separate review article [22].

To discover proteins bound to RNA of interest (RNA-centric methods), IVT (in vitro-transcribed) RNA can be tagged with biotin, and selectively bound to streptavidin for purification [23]. RaPID (RNA–protein interaction detection) [24] operates in a conceptually similar way to the previous method. IVT RNA can also be tagged with dyes and bound to protein microarrays, with fluorescence providing a quantitative output [25]. In vivo, cross-linking RNA with protein, either through formaldehyde or UV light, is used to identify LPI by purifying and extracting the RNA-bound proteins. CHART (capture hybridisation analysis of RNA targets) [26], ChIRP (chromatin isolation by RNA purification and capture hybridisation analysis of RNA targets) [27], MS2-BioTRAP (MS2 in vivo biotin-tagged RAP) [28], PAIR (peptide nucleic acid-assisted identification of RBPs) [29], RAP (RNA affinity purification) [30] and TRIP (tandem RNA isolation procedure) [31] all use either of these cross-linking strategies.

To discover RNA bound to proteins of interest (protein-centric methods), exploiting cross-linking is also common. The largest group of protein-centric methods are CLIP (cross-linking immunoprecipitation)-based methods [32]. Many variants of CLIP methods exist [33], and when paired with high-throughput sequencing are capable of generating libraries of data for further analysis. RIP-seq (RNA immunoprecipitation) [34] and TRIBE (targets of RNA-binding proteins identified by editing) [35] also belong to this category of protein-centric methods.

## 3. LncRNA-Protein Resource Databases

Starbase [36], POSTAR [37], RAIN [38], RNAInter [39], NPInter [40], ATtRACT [41] and oRNAment [42] are examples of databases that contain information associated with lncRNA-protein interactions obtained by the previously discussed laboratory assays, computational analysis and literature mining. Two broad classes exist: databases containing curated lncRNA-protein interactions and databases containing RNA-binding motifs.

Starbase, RNAInter, POSTAR, NPInter and RAIN all contain details of curated lncRNA-protein interactions, and many additional attributes (including functional annotation) associated with the interactions, derived from a combination of the laboratory assays discussed in the previous section (Table S1). These are not limited exclusively to lncRNA, and contain various other pieces of interaction information, including interactions with other ncRNA, other nucleic acids and proteins [43,44,45]. Some contrasts between these databases are also observable from a species, usability and scope perspective, which will be discussed here. Starbase, POSTAR and RAIN contain LPI information from a small number (two to four) of species, while RNAInter and NPInter host a wide range of species. To improve usability, Starbase, RNAInter and RAIN feature third party tool integration to streamline bioinformatics workflows. In terms of scope, POSTAR and NPInter appear to be focused on disease phenotypes, providing disease association information, while Starbase, RNAInter and RAIN have a more generic focus. 

ATtRACT and oRNAment databases contain details of RBP (RNA-binding protein) motifs. While not directly containing LPI, these can be applied to predict putative LPI and are a useful starting point or supplementary tool in screening for LPI.

To provide a guide for the community on database selection, we generated a recommendation matrix (Table S2). We considered five lncRNAs, namely NEAT1, MALAT1 and Hotair (well studied) versus Lassie and MaTAR25 (less explored). We discovered that Starbase is an exclusive database which provides MALAT1–protein interactions with the CLIP-seq evidence, whereas POSTAR2 provides RNA- and RBP-centric interactome information for the well-examined lncRNAs. Similarly, RAIN provides RNA–protein interaction details and networks using STRING for NEAT1, MALAT1 and HOTAIR. RNAInter provides information associated with interacting molecules, RNA editing, RNA structure, RNA localisation, RNA modification, evidence support (experimental evidence) and references, interaction networks (the top 100 interactions) and dynamic expression for the major lncRNAs. NPInter integrates NONCODE and ENSEMBL data to document and annotate the available information for NEAT1, MALAT1 and HOTAIR while ATtRACT is an RBP-centric database (keyword should be a RBP) that provides the RBP details and associated motifs. oRNAment consists of detailed information on transcripts and RBP along with numerous downloadable graphical representations of the noted lncRNAs with multiple visualisation options. However, none of these databases include any information on emerging lncRNAs such as Lassie and MaTAR25, further highlighting the reliance of the community on these databases.

All databases feature at least mouse and human datasets, likely due to their status as model organisms relevant to human disease, although some incorporate other model organisms as well. It is interesting to note that all databases feature advanced querying and search functions, likely reflecting the volume and complexity of LPI data. We have reviewed and compared them in Table S1. In summary, we discovered that there is a surprising lack of specialised LPI databases, with most databases featuring combinations of other nucleic acid and protein combinations. The biggest limitation of the current databases is that the LPI data are available only at a gene level and not a transcript level, lowering the resolution of LPI discovery methods which use these data. In a separate (unpublished) study we demonstrated that different isoforms of a lncRNA genes can have different interactomes, and hence functions. We are also developing machine learning methods to annotate lncRNAs at the transcript level (https://bioinformaticslab.erc.monash.edu/linc2function accessed on 27 May 2021).

## 4. LPI Prediction Algorithms

**Figure 1:**
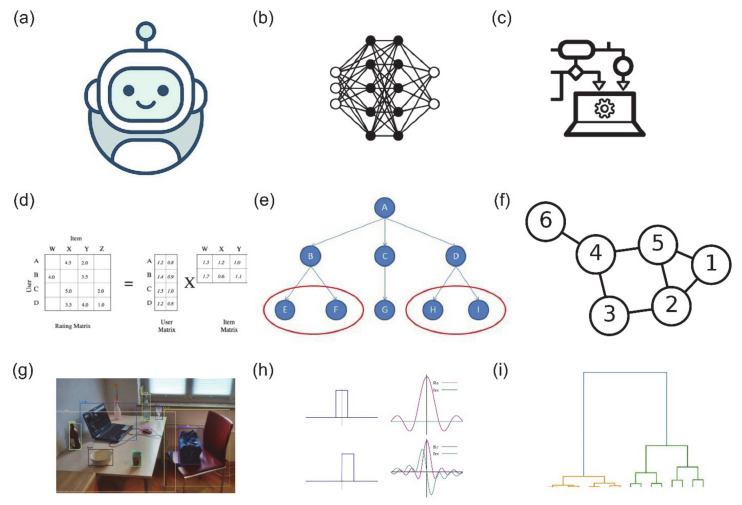
Visualisation of the broad categories of strategies used for predicting lncRNA-protein interactions. (**a**) Machine learning, (**b**) deep learning, (**c**) ensemble learning, (**d**) matrix factorisation, (**e**) similarity network analysis, (**f**) graph theory, (**g**) segmentation, (**h**) Fourier transform (in lncRNA-protein molecular docking simulations) and (**i**) hierarchical clustering. Training data are commonly higher-level features (e.g., structure, orientation) of lncRNA and proteins as well as the sequences recoded into tensors of varying dimensions.

Most LPI prediction algorithms exploit these curated databases of prior LPI knowledge to tune their predictions. Computational strategies for LPI prediction can be divided into two high-level categories, molecular docking and machine learning. Lower-level subdivisions among the methods we surveyed are visualised in Figure 1, and include deep learning, tree-based methods, graph-based methods, similarity networks, image segmentation, matrix factorisation and variants of the Fourier transform. Conventional molecular docking methods operate by finding the optimal configuration of an lncRNA-protein complex, and ranking the highest scoring configurations for further evaluation. Within the past decade, a large number of prediction algorithms based on machine learning have emerged. Most machine learning methods do not involve molecular docking simulations. Instead, they exploit known interactions between lncRNA and protein and/or biomolecular sequence information directly, although many also leverage known secondary structures to improve their performance table [1,2]. As with the LPI databases, it is worth noting that none of these methods are tuned specifically for LPI prediction, and represent broader scopes of identifying combinations of nucleic acid–protein interaction.

### 4.1. Molecular Docking Approaches

Before the current ecosystem of machine learning algorithms was established, molecular docking was the dominant strategy used to predict and investigate LPI or RNA–protein interactions in general. By developing custom equations, which account for conformation and other steric properties, the likelihood of lncRNA-protein complex formation is scored. Low-level methodology does not vary significantly, with most methods applying a variant of the FFT (fast Fourier transform) to extract features from three-dimensional molecule representations, template or optimising for a minimal energy state. Key factors considered include docking pose, distance and area of interracial sites, energy-based criteria and selection of the most structurally conserved docked complex [46]. Several methods also account for sequence homology or electrical charge between biological molecules [47]. Hierarchical clustering to group complexes of interest is not uncommon. However, at a high level these strategies are applied in different ways, and on different steric features. In many cases, a set of parameters must be specified by the user.

Most of the molecular docking methods we reviewed use methods which incorporate at least two of the previously discussed low-level methodologies (Table 1). To provide some context for the building blocks of these more complex methods, we first present examples of methods that use an individual strategy together with a brief discussion of their advantages and disadvantages, which include 3dRPC [48], HexServer [49], FireDOCK [50], HADDOCK [51] and PatchDOCK [52]. HexServer and 3dRPC are FFT-based methods, and 3dRPC is effective on well-characterised molecules only. By exploiting the fact that LPI complexes have looser packing, 3dRPC implements FFT on geometric complementarity as well as electrostatics with a custom scoring function. HexServer uses an FFT-based algorithm to exploit shape complementarity as a feature for optimisation. Its key advantage is its reformulation of the conventional 3D search space to greatly boost the speed of the FFT, achieving results in seconds. Meanwhile, FireDOCK and HADDOCK optimise the minimum free energy of the lncRNA-protein complex. While FireDOCK focuses on exploiting side chain information, HADDOCK leverages ambiguous interaction restraints, and is one of the few methods which has the advantage of being applicable to multi-body problems as well as other biomolecular interactions. Among molecular docking tools, PatchDOCK takes a more unconventional strategy by summarising low-level geometric features into higher-level features, and has some conceptual similarities to image segmentation. It is interesting to note that FireDOCK and PatchDOCK both complement each other, where PatchDOCK can feed output directly into FireDOCK.

Methods implementing a mixture of these strategies include HDOCK [53], MPRDOCK [54], P3DOCK [55] and NPDOCK [56]. HDOCK integrates template-based modelling as well as ab initio docking, with a scope that extends to both proteins and nucleic acids. In addition, the user may specify binding sites of interest directly. MPRDOCK exploits protein flexibility by applying FFT and considering sequence homology of the target of interest to generate a repertoire of structures for “ensemble docking”. We note that in this specific context of MPRDOCK, “ensemble docking” refers to the library of proteins generated by MPRDOCK, and is distinct from “ensemble learning” in the machine learning approaches section where the outputs of multiple algorithms are aggregated to obtain a result. P3DOCK integrates the previously discussed 3dRPC, as PRIME that leverages sequence as well as structural homology in addition to the features used by 3dRPC. P3DOCK’s authors claim that by complementing free docking and template-based docking strategies in a hybrid approach, a more accurate classification is possible. Finally, NPDOCK does not use a hybrid or ensemble strategy, but chains multiple methods into a pipeline of tools, which implement mostly FFT-based methods. The main advantage of using such ensemble methods is a generally improved performance over single-strategy methods as the limitations of each individual method are complemented. 

With the exception of one or two methods such as HexServer, many of these algorithms are computationally expensive and time-consuming (hours to days of real time) to run. Some methods, such as HexServer, require advanced hardware such as GPUs and specialised software engineering tools. Biological molecules are complex and dynamic, with their wide range of possible conformations as well as orientations greatly increasing the search space for algorithms. The molecular docking community is mindful of this, and provides their software on publicly accessible and user-friendly web servers for users to run these programs remotely, although time remains a bottleneck for these workflows.

### 4.2. Machine Learning Approaches

Most modern lncRNA-protein interaction (LPI) prediction algorithms use machine learning, where large datasets with attributes of interest are passed to an algorithm (Table 2). The algorithm then “learns” from the data, discovering patterns in the data with minimal human intervention such as user-defined equations, a process known as “training”. In the case of LPI, known LPI and their corresponding sequences as well as structures are used for training the prediction models. Their strategies can be divided into several broad categories, including graph methods, ensemble learning, matrix factorisation and deep learning. Of these strategies, matrix factorisation appears to be the most popular and is integrated into many other higher-level strategies. LPI are commonly formulated as similarity matrices, which can then be easily formulated as a matrix factorisation problem. Broader strategies incorporating matrix factorisation, such as ensemble learning and methods which leverage multimodal data, appear to have consistently robust performance [57]. Few deep learning models exist, but they both perform and generalise well in comparison to other methods, and are likely to become more popular as they have become in other areas of biology.

Matrix factorisation is the most common way to formulate LPI for prediction algorithms, including LPI-FKLKRR (lncRNA-protein interaction kernel ridge regression, based on fast kernel learning) [58], LPI-KTASLP (prediction of lncRNA-protein interaction by semi-supervised link learning with multivariate information) [59], LPI-NRLMF (lncRNA-protein interaction prediction by neighbourhood regularised logistic matrix factorisation) [60], LPI-INBRA (long non-coding RNA–protein interaction prediction based on improved bipartite network recommender algorithm) [61] and LPI-BNPRA (long non-coding RNA–protein interaction bipartite network projection recommended algorithm) [62]. These methods share a common theme of formulating lncRNA-protein interactions as a matrix factorisation problem and using them in broader strategies, such as multiple kernel learning or recommender algorithms. Known structural features are often used together with sequence features. In the special case of LPI-FKLKRR, matrices are reformulated into kernels for direct optimisation with kernel ridge regression, increasing performance in the common scenario of class imbalance. Further comparing and contrasting the advantages as well as disadvantages of these methods shows that LPI-FKLKRR and LPI-KTASLP are expected to be effective in the case of imbalanced classes. In LPI-NRLMF, the authors note a slight prediction bias may occur due to the sparsity of their training data. LPI-INBRA is robust against false positives, and LPI-BNPRA is effective on closely related species other than humans.

Some graph-based methods for LPI prediction are PBLPI (path-based lncRNA-protein interaction) [63] and PLPIHS (predicting lncRNA-protein interactions using HeteSim scores) [64]. PBLPI takes into account both functional and semantic similarity between proteins, while PLPIHS uses a custom distance metric to unify co-expression, lncRNA-protein interactions and protein–protein interaction scores to construct a network which is then provided to a SVM classifier. In the case of PBLPI, a disadvantage is that prediction accuracy may be reduced due to technical limitations, while in PLPIHS performance is improved by preserving information regarding the biological network, taking into account lncRNA-protein interactions similar to the target. 

Examples of hybrid and ensemble learning approaches are IRWNRLPI (integrating random walk and neighbourhood regularised logistic matrix factorisation for lncRNA-protein interaction prediction) [65], SFPEL-LPI (sequence-based feature projection ensemble learning method) [66], HLPI-Ensemble (human lncRNA-protein interactions ensemble) [63], GPLPI (graph predict lncRNA-protein interaction) [67], LPLNP (linear neighbourhood propagation method) [68] and LPI-BLS (predicting lncRNA-protein interactions with a broad learning system-based stacked ensemble classifier) [69]. IRWNRPLI uses lncRNA-protein interactions and lncRNA/protein sequence similarity as the input into a hybrid approach of random walk and neighbourhood regularised logistic matrix factorisation. Being an integrative model, it appears to be robust, although its accuracy varies on different biological systems. Ensemble approaches PMKDN, SFPEL-LPI, HLPI-Ensemble, LPI-BLS and LPLNP all have the advantage of being robust against noise due to their ensemble strategy, incorporating multiple approaches, and are capable of discovering new LPI. LPLNP and LPI-BLS in particular stand out: LPI-BLS for its unconventional flat network architecture and aggregation strategy, as well as its effectiveness in multiple species, and LPLNP for its unique application of neighbourhood similarity to LPI. However, we note that HLPI-Ensemble is specifically intended for human LPI only. GPLPI uses both sequence features and known secondary structures to train a graph-based neural network. In addition, by using an ensemble of features including evolutionary information, GPLPI’s effectiveness was increased. An important distinction between these two methods is that GPLPI is trained on known plant lncRNA, and plant non-coding RNA have different properties (some ncRNA lose function even with 1–2 nucleotide changes) to those of animal non-coding RNA [70]. For this model to be effective on non-plant organisms, retraining is likely necessary but viable due to the relatively higher volume of data associated with animals, in particular humans [63].

Only a few deep learning approaches exist: DeepBind [70], LPI-CNNCP (lncRNA-protein interactions convolutional neural network copy-padding trick) [71] and DeepLPI (deep lncRNA-protein interactions) [72]. DeepBind was one of the first applications of deep learning to predict nucleic acid–protein binding, and is applicable to LPI. By reformulating the classical position weight matrix [73] as a convolutional kernel, it operates on raw sequence data to provide a simple prediction score for a nucleic acid–protein interaction [74]. LPI-CNNCP uses only lncRNA and protein sequence data recorded as k-mers as input into a CNN but achieves good results. It is also interesting to note that it appears to be one of the few models that are effective across different species, which is a less common advantage. Meanwhile, DeepLPI feeds co-expression, sequence and structural data to a neural network optimised by a conditional random field. Using isoform data makes DeepLPI the only method to date with the ability to predict lncRNA interaction with different protein isoforms. Furthermore, its flexibility allows it to be extended to other biomolecular interactions, such as miRNA.

Other methods used to predict LPI that do not fall into a specific category include LPI-SKF (lncRNA-protein interaction similarity kernel fusion) [75], PMKDN (projection-based neighbourhood non-negative matrix decomposition model) [73] and LPI-MiRNA [74]. LPI-SKF uses an integrative approach where verified lncRNA-protein interactions are used to build a network, and similarity kernel fusion is used to integrate protein and lncRNA similarity scores before applying manifold learning. PMKDN uses multiple features from lncRNA (nucleotide composition, expression levels) and protein (amino acid subcategories) to build a similarity matrix for similarity network fusion with a nearest neighbour’s approach. Both these methods have the advantages of being robust against noise and capable of interaction discovery, but like most methods that express LPI as similarity matrices, they make a strong assumption that sequence homology correlates with interactivity, which may not hold in all cases. LPI-MiRNA takes a unique approach, exploiting miRNA as an intermediate unit of lncRNA-protein binding, and uses this in a network-based approach. While this gives LPI-MiRNA the ability to operate on datasets without prior knowledge of lncRNA interactions, a different limitation is introduced of relying on known miRNA–lncRNA and miRNA–protein interactions. An assumption is also made that miRNAs which interact with both lncRNA and a protein would also form LPI, which may not always hold. Nevertheless, this method was shown to be effective. 

Although lncPro [76] and catRAPID [77] are older methods, these are featured in this manuscript because of their historical significance. lncPro was one of the first published machine learning LPI prediction algorithms, and many LPI algorithms resemble it. Higher-level features are extracted from lncRNA and protein sequence, which are then recorded as vectors as input into their model. Although the authors noted limitations associated with data availability and computational complexity at the time, this method became a template for many other machine learning methods, including those discussed in this manuscript. catRAPID does not apply machine learning, but instead constructs an interaction matrix from known secondary structure and other molecular features. A major limitation of this approach is its reliance on obsolete genomic data, which is expected to reduce prediction accuracy.

However, it is important to note that the scope of most LPI prediction algorithms are limited. Not all methods can predict interactions for novel lncRNA or proteins, and few methods generalise across species [62,69,71]. This is partly due to the limited availability of curated training data, with a small number of samples mostly from human or mouse present in a few databases [63,66,69]. LPI prediction for different protein isoforms is also not an active area of prediction algorithm development, with only one method having this functionality. Another limitation observed is that some methods exploit sequence similarity as an intermediate metric for LPI prediction, particularly methods which formulate LPI as similarity matrices. While this appears to be effective within the specific training datasets used by each study, this implicit assumption of similar sequence homology correlating to interactivity may not always hold, especially across different species [78,79]. At the same time, we consider that small nucleotide changes in biological molecules can cause major functional changes, which can potentially cause improperly trained prediction algorithms to produce misleading results [80]. 

We also note the limited accessibility of many of these machine learning methods. Among the methods reviewed that were published within the last five years, many do not make their source code publicly available and/or are written in proprietary programming languages such as MATLAB [81]. This restricts reproducibility and prevents usage of more than half of the methods we reviewed (Table 2). At least partly because of the computational complexity required, machine learning methods which are well suited to resolving non-linear variables in high dimensional data have recently become a focus of the LPI field. Computational methods that both identify and functionally annotate LPI are limited, leaving a gap in the field. 

In contrast to published molecular docking algorithms, only a few machine learning methods provide active web servers for convenient use by the community, further raising the barrier for usability by biologists.

## 5. Future Directions

Computational surveying is not a substitute for experimental validation. However, as the intention of computational modelling is to generate a subset of the most likely testable hypotheses for laboratory biologists, we believe that developments in both the laboratory and computational fields will complement each other. With computational modelling reducing the quantity of experiments required, and with the experimentally validated data generated as a result, more efficient algorithms can be developed which further reinforces the developmental cycle. As a result, biologists interested in LPI will gain access to more refined tools, allowing them to streamline their experiments.

## 6. Conclusions

LPI forms a unique layer of gene regulation across many species, and a growing interest in the field has resulted in the creation and expansion of curated databases as well as LPI prediction algorithms. Here, we are reviewing some of the established (older than five years) and recent (within the last five years) LPI prediction approaches as well as databases. We note four important points. First, there has been a recent shift from conventional molecular docking algorithms to machine learning methods, which attempt the direct prediction of LPI from biomolecular sequence identity and higher-level features. This shift to machine learning is observable across different fields of biology and is likely to continue with the rising availability of computational infrastructure as well as machine learning expertise. Secondly, these methods are heavily dependent on a set of curated data across several databases. Across these databases, a lack of universal standardisation complicates data merging [82], preventing the community from unlocking the full potential of LPI data, in contrast to conventional transcriptomics databases such as SRA [83], EBI [84] and DDBJ [85]. This is in part due to the diversity of assays used to capture the LPI information, as well as the scope of the databases, which may subsequently bias the machine learning algorithms developed on these data. Third, there is a distinct lack of methods and databases which are specifically designed for LPIs’ unique properties, with most having a generic scope despite LPIs’ biological significance. Finally, it is concerning that more than half of the recent machine learning methods we surveyed are not reproducible or usable due to the absence of or restrictions on their source code. However, LPI act as an important but less-studied regulatory layer and understanding them will provide key context to deepen our understanding of biological systems.

## Figures and Tables

**Table 1:** A comparison of molecular docking tools used to predict lncRNA-protein interactions. Important attributes of these molecular docking tools, including their effectiveness and a link to their corresponding server, are listed (all weblinks are accessed on 27 May 2021).

Sl:No	Resource	Resource Type	Advantages and Disadvantages	Weblink	Reference Paper
1	P3DOCK	LncRNA–protein docking server (adapted from conventional docking servers)	Free docking and template-based docking strategies in a hybrid approach, results in an accurate classification	http://www.rnabinding.com/P3DOCK/P3DOCK.html	[55]
2	HDOCK	LncRNA–protein docking server (adapted from conventional docking servers)	Integrates template-based modelling as well as ab initio free docking, with a scope that extends to both proteins and nucleic acids	http://hdock.phys.hust.edu.cn	[53]
3	PATCHDOCK	LncRNA–protein docking server (adapted from conventional docking servers)	Low-level geometric features into higher-level features, FireDOCK and PatchDOCK both complement each other, where PatchDOCK can feed output directly into FireDOCK.	https://bioinfo3d.cs.tau.ac.il/PatchDock/ /	[52]
4	FIREDOCK	LncRNA–protein docking server (adapted from conventional docking servers)	Focuses on exploiting side chain information, optimises the minimum free energy of the lncRNA-protein complex	http://bioinfo3d.cs.tau.ac.il/FireDock/ /	[50]
5	NPDOCK	Exclusively lncRNA-protein docking server, developed for nucleic acid docking only	Chains multiple methods into a pipeline of tools, which implement mostly FFT-based methods.	http://genesilico.pl/NPDock /	[56]
6	HADDOCK	LncRNA–protein docking server (adapted from conventional docking servers)	It averages ambiguous interaction restraints, and it can generalise to multi-body problems as well as other biomolecular interactions, optimises the minimum free energy of the lncRNA-protein complex	https://wenmr.science.uu.nl/haddock2.4/	[51]
7	MPRDOCK	LncRNA–protein docking server (adapted from conventional docking servers)	Implies protein flexibility by applying FFT and considering sequence homology of the target of interest to generate a repertoire of structures for “ensemble docking”	http://huanglab.phys.hust.edu.cn/mprdock/	[54]
8	Hexserver	LncRNA–protein docking server (adapted from conventional docking servers)	FFT-based algorithm to exploit shape complementarity as a feature for optimisation	http://hexserver.loria.fr/	[49]

**Table 2:** A comparison of machine learning algorithms used to predict lncRNA-protein interactions. Important attributes of these machine learning algorithms, including their scope, strategies, training data, effectiveness and reproducibility are listed. More than half of these methods are not reproducible as their source code is proprietary or not available. A few methods provide web interfaces for users to enter their own data (all weblinks are accessed on 27 May 2021).

Sl:no	Resource	Scope	Advantages and Disadvantages	Strategy	Problem Formulation	Model Training Data	Weblink/Source Code	Reference Paper
1	LPI-FKLKRR (lncRNA-protein interaction kernel ridge regression, based on fast kernel learning)	Prediction	Effective in datasets with imbalanced classes.	Kernel ridge regression	Similarity matrices formulated as kernels	lncRNA-protein interactions, lncRNA expression, protein ontology, lncRNA sequence, protein sequence	https://github.com/6gbluewind/LPI_FKLKRR	[58]
2	LPI-KTASLP (prediction of lncRNA-protein interaction by semi-supervised link learning with multivariate information)	Prediction, discovery	Effective in datasets with imbalanced classes.	Multiple kernel learning	Similarity matrices formulated as kernels	lncRNA-protein interactions, lncRNA expression, lncRNA sequence	https://github.com/6gbluewind/LPI_KTASLP	[59]
3	LPI-NRLMF (lncRNA-protein interaction prediction by neighbourhood regularised logistic matrix factorisation)	Prediction, discovery	Prediction bias is expected due to the sparsity of the training dataset.	Matrix factorisation	Similarity matrices	lncRNA-protein interactions, lncRNA sequence, protein sequence	NA	[60]
4	LPI-INBRA (long non-coding RNA–protein interaction prediction based on improved bipartite network recommender algorithm)	Prediction	Robust against false positives.	Matrix factorisation	Similarity matrices	lncRNA-protein interactions, lncRNA sequence, protein sequence	NA	[61]
5	LPI-BNPRA (long non-coding RNA–protein interaction bipartite network projection recommended algorithm)	Prediction	Effective in humans and closely related species.	Bipartite network recommendation	Similarity matrices	lncRNA-protein interactions, lncRNA sequence, protein sequence	NA	[62]
6	PBLPI (path-based lncRNA-protein interaction)	Prediction, discovery	Prediction accuracy limited due to technical limitations.	Graph	Similarity matrices	lncRNA-protein interactions, protein semantic similarity, lncRNA functional similarity, Gaussian interaction profile kernel similarity, integrated similarity for lncRNAs and proteins	NA	[63]
7	PLPIHS (predicting lncRNA-protein interactions using HeteSim scores)	Prediction, discovery	Performance is improved by preserving information regarding the biological network, taking into account lncRNA-protein interactions similar to the target.	Graph	Similarity matrices	Co-expression data of lncRNA-protein pairs, lncRNA-protein interaction data	NA	[64]
8	IRWNRLPI (integrating random walk and neighbourhood regularised logistic matrix factorisation for lncRNA-protein interaction prediction)	Prediction	Robust due to hybrid approach, but known to be unstable.	Hybrid: random walk, neighbourhood regularised logistic matrix factorisation algorithm	Similarity matrices	lncRNA-protein interactions, lncRNA sequence, protein sequence	NA	[65]
9	SFPEL-LPI (sequence-based feature projection ensemble learning method)	Prediction, discovery	Multimodal approach boosts prediction accuracy.	Ensemble: graph Laplacian regularisation	Similarity matrices	lncRNA-protein interactions, lncRNA sequence, protein sequence	http://www.bioinfotech.cn/SFPEL-LPI/	[66]
10	HLPI-Ensemble (human lncRNA-protein interactions ensemble)	Prediction	Scope restricted to humans.	Ensemble: support vector machines (SVM), random forests (RF) and extreme gradient boosting (XGB)	Recoded feature vectors	lncRNA-protein interactions, lncRNA sequence, lncRNA features, protein sequence, protein features	NA	[63]
11	GPLPI (graph predict lncRNA-protein interaction)	Prediction	Scope restricted to plants.	Deep learning, ensemble learning, graph attention LSTM autoencoder	Recoded sequence and structure vectors	lncRNA sequences, protein sequences, structural features from predicted secondary structures from lncRNA and protein sequences.	https://github.com/Mjwl/GPLPI	[67]
12	LPI-BLS (predicting lncRNA-protein interactions with a broad learning system-based stacked ensemble classifier)	Prediction	Flat network architecture boosts speed and accuracy. Effective in several model organisms.	Ensemble: broad learning system (flat neural network)	Recoded feature vectors	lncRNA-protein interactions, lncRNA sequence, lncRNA features, protein sequence, protein features	https://github.com/NWPU-903PR/LPI_BLS	[69]
13	LPI-CNNCP (lncRNA-protein interactions convolutional neural network copy-padding trick)	Prediction	Can be extended to predict other biomolecular interactions, effective across different species.	Deep learning (convolutional neural network)	Recoded feature vectors	lncRNA-protein interactions, lncRNA sequence, protein sequence	https://github.com/NWPU-903PR/LPI-CNNCP	[71]
14	DeepLPI (deep lncRNA-protein interactions)	Prediction, discovery	Can be extended to other biomolecular interactions, unique capability to predict lncRNA interaction with different protein isoforms.	Deep learning (embedding, convolution, LSTM)	Recoded feature tensors	lncRNA-protein interactions, lncRNA sequence, lncRNA structure, protein sequence, protein structure	https://github.com/dls03/DeepLPI	[72]
15	LPI-SKF (lncRNA-protein interaction similarity kernel fusion)	Prediction, discovery	Aggregating multiple similarities increases robustness against noise.	Similarity kernel fusion, manifold learning	Similarity matrices	lncRNA-protein interactions, pairwise similarities for lncRNAs, pairwise similarities for proteins	https://github.com/zyk2118216069/LPI-SKF	[75]
16	PMKDN (projection-based neighbourhood non-negative matrix decomposition model)	Prediction	Strategy avoids overfitting and sparsity issues, allowing more generalisability to different datasets.	Neighbourhood regularised matrix factorisation algorithm	Similarity matrices	lncRNA-protein interactions, lncRNA sequence, lncRNA expression, protein sequence, protein annotation	NA	[73]
17	LPI-miRNA	Prediction, discovery	Can operate on datasets without prior knowledge of lncRNA interactions but relies on known miRNA–lncRNA and miRNA–protein interactions.	Heterogeneous network model	Similarity matrices	lncRNA–miRNA interactions, protein–miRNAs interactions	https://github.com/zyk2118216069/LncRNA-protein-interactions-prediction	[74]
18	lncPro	Prediction	Training dataset limited, effective on short sequences.	Fourier transform, matrix factorisation	Recoded feature tensors	lncRNA-protein interactions, lncRNA sequence, lncRNA features, protein sequence, protein features	http://cmbi.bjmu.edu.cn/lncpro/	[76]
19	catRAPID	Prediction	Visualisation is available, prediction accuracy may be limited by reliance on very old lncRNA annotations.	Discrete Fourier transform	lncRNA and protein secondary structure, hydrogen bonding, van der Waals forces	NA	http://s.tartaglialab.com/page/catrapid_group	[77]
20	3dRPC	Prediction	Effective on well-characterised molecules, may have lower accuracy if this is not the case.	Fast Fourier transform, root mean square deviation	Conformations of nucleotide-amino-acid pairs	NA	http://biophy.hust.edu.cn/3dRPC.html	[48]
21	DeepBind	Prediction	Effective, generalisable across species, but more effective at predicting protein–DNA binding than protein–RNA binding.	Deep learning (convolutional neural network)	Recoded feature tensors	lncRNA-protein interactions, lncRNA sequence, protein sequence	http://tools.genes.toronto.edu/deepbind/	[70]
22	LPLNP	Prediction, discovery	Effective and robust in humans, capable of discovering novel interactions.	Ensemble: linear neighbourhood similarity	Similarity matrices	lncRNA expression, lncRNA features lncRNA-protein interactions, lncRNA sequence, protein features, protein sequence	https://github.com/BioMedicalBigDataMiningLabWhu/lncRNA-protein-interaction-prediction	[68]

## Supplementary Materials

The following are available online at https://www.mdpi.com/article/10.3390/ncrna7020033/s1. Table S1: LncRNA–protein data repositories. Seven databases, four with LPI information and three with RNA motif information, are surveyed. Each database holds information on at least one combination of nucleic acid and protein interaction. The number of species each database contains varies widely, from 4–154. Every database contains at least human and mouse data, and has been updated within the past five years, Table S2: LPI database recommendation matrix. Seven databases are analysed with respect to lncRNAs, namely, NEAT1, MALAT1 and Hotair (well studied) versus Lassie and MaTAR25 (less explored). Each database includes information on NEAT1, MALAT1 and Hotair and there are no data available regarding Lassie and MaTAR25.
